# Death of the Foot Produced by Tight Bandages

**Published:** 1858-06

**Authors:** 


ART. V.—*Death of the Foot Produced by Tight Bandages.*

Dr. Flint: The following case has been communicated to me by Dr. Fuller, of Wyoming, with permission to publish it. Examples of death of the hand and arm from tight bandages are not unfrequent, but I have found upon record no other case of death of the foot from the same cause.

Frank H. Hamilton.

A. B., of C., æt. 71. Fell from a tree, striking upon his foot, Aug. 27, 1855, producing a backward dislocation of the tibia and fibula upon the os calcis, and also a fracture of both bones of the leg a few inches above.

An eclectic physician took charge of him, applying lateral splints, and a roller firmly from the toes to the knees. Notwithstanding the remonstrances and prayers of the patient to have the bandage loosened, it was kept on until the 9th day, when the doctor cut the bandage upon the top of the foot. It was then vesicated about the ankle; simple cerate was applied, but the remainder of the bandage was continued. No further change was made until the 19th of Sept., when I was called in. On removing the dressings, I found the integuments covering the whole foot, dead and dried down to the bones. The dislocation was not reduced. Soon after the whole limb became œdematous, and on the 27th of Oct. the leg was amputated by Dr. Barrett, of Leroy. The patient recovered rapidly.

